# Navigating Japan’s COVID-19 Vaccination Challenges: A Shift in Legal Classification and the Opt-out System

**DOI:** 10.31662/jmaj.2023-0061

**Published:** 2023-11-27

**Authors:** Yudai Kaneda, Mira Namba, Masaki Takebayashi

**Affiliations:** 1 School of Medicine, Hokkaido University, Hokkaido, Japan; 2 School of Medicine, Keio University, Tokyo, Japan; 3 Graduate School of Aomori University of Health and Welfare, Aomori, Japan

**Keywords:** COVID-19, Vaccination Coverage, Opt-Out, Infectious Disease Control Act, Japan

## Abstract

On January 20, 2023, the Japanese government announced easing the legal handling of the novel coronavirus disease 2019 (COVID-19) under its Infectious Diseases Control Act, effective May 8, 2023. While free vaccines will continue in fiscal year 2023, the future of mass vaccinations remains uncertain. The opt-out system, wherein local governments schedule vaccinations on behalf of the residents, may potentially alleviate issues associated with the currently adopted opt-in approach, such as procedural intricacies and scheduling difficulties, thereby facilitating the recovery of vaccination rates and simultaneously addressing vaccine wastage concerns. Given that COVID-19 still presents a substantial risk to specific groups, such as the elderly, recognizing the benefits of the opt-out system and the ethical and geographical challenges it poses is essential. With the collaboration of local governments and healthcare institutions, ongoing surveillance and scientific assessment are indispensable.

On January 20, 2023, the Japanese government announced that commencing May 8, 2023, management of the novel coronavirus disease 2019 (COVID-19) will be relaxed under the Infectious Diseases Control Act (IDCA) based on the scientific evidence that the Omicron variant has lower severity and mortality rates compared with previous strains ^(1)^. This act categorizes infectious diseases into five classes based on infection and severity. COVID-19 was previously categorized as pandemic influenza and managed more strictly than Class 2 infectious diseases due to its high potential of causing significant harm to human health; the implementation of measures such as travel restrictions for infected individuals, investigation of infection routes, and contact tracing was initiated. However, with this decision of the government, it would be shifted to a Class 5 infectious disease, aligned with seasonal influenza, which is considered significantly less risky to human health. In this context, COVID-19 vaccines, supported by substantial evidence of their infection-controlling efficacy, were offered free of charge as exceptional temporary vaccinations under the Immunization Act of Japan, given the urgent need to curtail the spread of the disease. However, with changing legal classification and the potential diminished sense of urgency, the duration of the free vaccine provision, although announced by the Japanese Ministry of Health, Labor, and Welfare (MHLW) to continue for the fiscal year 2023, remains uncertain.

In Japan, COVID-19 vaccinations are currently conducted as mass vaccinations under the supervision of each municipality. However, considering that not enough clinics would be equipped with the necessary refrigeration facilities for proper vaccine storage, mass vaccinations could not be replaced with individual vaccinations. In addition, since COVID-19 vaccines currently used in Japan, manufactured by Pfizer and Moderna, come in one-vial sets for 6-10 individuals ^(2)^, it may be challenging to gather enough applicants each time at each clinic, as the number of people wanting to be vaccinated is decreasing. Indeed, multiple institutions have reported instances of vaccine waste due to refrigeration storage and reservation management ^(3)^. While the introduction of bivalent vaccine has now permitted a refrigerated storage period of up to 10 weeks, engendering optimism for an improved situation, it is estimated that vaccine wastage in Japan exceeds 80 million doses, with the actual state yet to be fully understood. Viewing the anticipated challenges associated with vaccine expiration in the future, there is an urgent need for comprehensive, nationwide interventions.

Furthermore, it is debatable that most Japanese municipalities have adopted the opt-in system as the COVID-19 vaccination framework. This system necessitates eligible individuals to proactively reserve their vaccination appointment, specifying the date, time, and location. Nevertheless, the complexity of scheduling and procedural logistics associated with the opt-in system has been identified as a barrier to vaccination, potentially resulting in individuals not receiving the vaccine despite their intentions ^(4)^. A decline in vaccination rates not only escalates the risk of severe disease post infection but also exacerbates vaccine wastage ^(3)^. Recognizing that a shift in legal classification does not denote an end to the pandemic, it is imperative to emphasize the ongoing need for robust vaccination efforts.

A potential solution to mitigate these concerns can be an opt-out system implementation, where local governments arrange vaccination appointments on behalf of the residents. This strategy could offer improved control over vaccine distribution, as the number of individuals to be vaccinated can be ascertained in advance, alleviating issues such as vaccine wastage. Indeed, the application of opt-out systems has proven to be effective across a variety of healthcare policy settings. A prior study conducted in the United States concerning influenza vaccination among university staff reported a 45% uptake rate under opt-out conditions, compared with 33% under opt-in conditions ^(5)^. The study concluded that an opt-out system increased the likelihood of scheduling influenza vaccinations, enhancing the probability of receiving the vaccination ^(5)^. Similarly, a study from Turkey reported a 13%-18% rise in deceased organ donation rates after the introduction of an opt-out system ^(6)^. This reform helped overcome formal legal requirements for family consent and the maintenance of written procurement criteria for deceased donors ^(6)^. Additionally, concerning the COVID-19 vaccine, a study from China indicated that the introduction and improvement of opt-out policies have successfully augmented individuals’ willingness toward vaccination ^(7)^.

However, to our knowledge, there is not enough information on the actual effectiveness of introducing an opt-out system on COVID-19 vaccination rates. While many municipalities adopted an opt-in approach, a few, such as Minamisoma City in Fukushima Prefecture and Mutsu City in Aomori Prefecture, adopted an opt-out system. In fact, since December 2022, Minamisoma City boasted the highest vaccination rate among all 815 municipalities in Japan at 69.1% ^(8)^. Impressively, the vaccination rate for the Omicron variant in Mutsu City was reported at 71.5% by mid-February 2023, markedly surpassing the national average of 42.9% during the same period ^(9),(10)^. Given this, there is a need for cooperation between administrative and medical experts to reflect on vaccination rates and systems and validate whether an opt-out policy is effective for better pandemic countermeasures in the future.

Even if the opt-out system proves to be effective, Japanese municipalities have diverse characteristics in terms of population size, age distribution, area, and transportation infrastructure; at present, not all municipalities can adopt the opt-out system. Younger generations, particularly in urban areas, may have difficulty receiving vaccinations through the opt-out system due to factors such as remote workplaces and weekday work schedules. Moreover, any implementation of an opt-out system for COVID-19 vaccination would require consideration of ethical and social issues related to individual rights ^(11)^. Historically, as seen in cases such as the smallpox vaccine, measures prioritizing the larger public welfare can be justified, especially concerning public health crises caused by infectious diseases ^(12)^. However, if the mortality rate from COVID-19 is deemed sufficiently low due to acquired immunity or the development of effective treatments, adopting an opt-out system could become debatable. Therefore, in implementing an opt-out system, it is crucial to emphasize that vaccination is an individual right and no inconveniences should arise from the cancellation of vaccination in information dissemination. In addition, to appropriately assess these situations and reflect them in policies, continuous surveillance of the infection situation and ongoing scientific evaluations are essential, especially after the declaration of the end of the pandemic. Furthermore, conducting such scientific verifications can prepare for timely and appropriate responses to the potential threats of novel infections, such as avian influenza and other anticipated novel infections.

In conclusion, the implementation of an opt-out system in the COVID-19 vaccination strategy, despite presenting ethical and geographical challenges, can potentially address issues such as the intricacy of the opt-in system’s processes, current low vaccination rates, and vaccine wastage. While the fatality rate from COVID-19 infections among the Japanese population under 60 has reportedly decreased from 0.08% with the Delta variant to <0.01% with the mainstream Omicron variant, according to MHLW, it continues to pose a significant risk for older adults and those with underlying medical conditions ^(1)^. Instead of focusing predominantly on the disease’s classification under IDCA, it is critical to assess effective vaccine distribution methods and contemplate vaccination policies tailored to the circumstances of each municipality, particularly for populations at high risk. Even following the declaration of the pandemic’s conclusion, the unpredictable timeline for the complete COVID-19 eradication necessitates that administrative and healthcare professionals continue their cooperative endeavors, actively implementing and systematically verifying public health measures.

## Article Information

### Conflicts of Interest

None

### Author Contributions

Conception, designing the study, and writing this paper; KY

Critical revision of the paper; NM and TM

All the authors read the final draft and approved its submission.
